# Advances on Liquid Biopsy Analysis for Glioma Diagnosis

**DOI:** 10.3390/biomedicines11092371

**Published:** 2023-08-24

**Authors:** Panagiotis Skouras, Mariam Markouli, Theodosis Kalamatianos, George Stranjalis, Penelope Korkolopoulou, Christina Piperi

**Affiliations:** 1 Department of Biological Chemistry, Medical School, National and Kapodistrian University of Athens, 11527 Athens, Greece; panosskouras@hotmail.gr; 2 1st Department of Neurosurgery, Evangelismos Hospital, National and Kapodistrian University of Athens, 11527 Athens, Greece; tkalamatian@med.uoa.gr (T.K.); gstranjalis@med.uoa.gr (G.S.); 3 Department of Medicine, Boston Medical Center, Boston University School of Medicine, Boston, MA 02118, USA; myriam.markouli@bmc.org; 4 Department of Pathology, Medical School, National and Kapodistrian University of Athens, 75 M. Asias Street, 11527 Athens, Greece; pkorkol@med.uoa.gr

**Keywords:** glioma, glioblastoma, tissue biopsy, liquid biopsy, cfDNA, circulating tumor cells, exosomes, LINE-1, epigenetics, radiogenomics

## Abstract

Gliomas comprise the most frequent primary central nervous system (CNS) tumors, characterized by remarkable genetic and epigenetic heterogeneity, difficulty in monitoring, and increased relapse and mortality rates. Tissue biopsy is an established method of tumor cell collection and analysis that enables diagnosis, classification of different tumor types, and prediction of prognosis upon confirmation of tumor’s location for surgical removal. However, it is an invasive and often challenging procedure that cannot be used for frequent patient screening, detection of mutations, disease monitoring, or resistance to therapy. To this end, the minimally invasive procedure of liquid biopsy has emerged, allowing effortless tumor sampling and enabling continuous monitoring. It is considered a novel preferable way to obtain faster data on potential tumor risk, personalized diagnosis, prognosis, and recurrence evaluation. The purpose of this review is to describe the advances on liquid biopsy for glioma diagnosis and management, indicating several biomarkers that can be utilized to analyze tumor characteristics, such as cell-free DNA (cfDNA), cell-free RNA (cfRNA), circulating proteins, circulating tumor cells (CTCs), and exosomes. It further addresses the benefit of combining liquid biopsy with radiogenomics to facilitate early and accurate diagnoses, enable precise prognostic assessments, and facilitate real-time disease monitoring, aiming towards more optimal treatment decisions.

## 1. Introduction

Gliomas comprise the most frequent primary tumor of the central nervous system (CNS), characterized by increased heterogeneity and aggressiveness, as well as high relapse and mortality rates [1]. More specifically, gliomas are responsible for 24.7% and 74.6% of all primary and malignant brain tumors, respectively [2]. Glioblastoma (GB), the most malignant glioma subtype is a highly aggressive, infiltrating, and poorly managed brain tumor. Currently available therapeutical approaches include surgical resection, followed by radiotherapy, as well as temozolomide (TMZ) chemotherapy. Thus far, limited drug penetration to the CNS, along with the rapid development of chemotherapy resistance, present ongoing challenging issues that contribute to poor prognosis [3,4].

Until now, tissue sample acquisitions from biopsies have been used for histological and molecular tumor analysis to confirm diagnosis, classify tumor types, detect tumor-specific mutations, and guide therapeutic protocols [5]. Tissue biopsy, although of major importance, is, however, an invasive and oftentimes challenging procedure. A surgical brain tissue biopsy increases the risk of bleeding and the likelihood of damaging an important brain area (e.g., glioma infiltrating a sensitive area of the frontal lobe), which can lead to neurological deficits, especially in the cases of the developing pediatric brain [6]. 

Moreover, invasive procedures are characterized by limitations in acquiring tumor samples in both efficient quantity and quality of tumor material. Sequential collection of tissue biopsies during treatment to monitor tumor response and relapse also poses a major challenge in tumor profiling [7]. It often provides limited material to perform all necessary molecular tests that are required to achieve a reliable diagnosis. In this context, the detection of mutations, genetic and epigenetic defects, or polymorphisms is not always possible.

In addition, tissue biopsy may not enable satisfactory detection of early-stage tumor or residual lesions and is not suitable for screening [8]. Furthermore, temporal and spatial tumor heterogeneity may decrease the practical utility of tissue biopsies to be used as tools for monitoring tumor progression and evaluating response to therapy [5]. In multifocal cases in particular, multiple biopsies may be necessary in order to obtain an accurate representation of the tumor, since invasive tumors constantly evolve both spatially and temporally over time or in response to treatment [7]. 

To this end, the minimal invasive procedure of liquid biopsy has surfaced as a solution to these issues, constantly progressing to enable first tumor diagnosis and collecting information on risk, prognosis, and recurrence potential. 

In this review, we describe recent advances on liquid biopsy analysis for glioma patients, pointing towards the most promising biomarkers that can be used to explore tumor characteristics. Moreover, the combination of liquid biopsy with radiogenomics is suggested not only as a potential approach to achieve early and accurate diagnoses and prognostic assessments but also to enable real-time disease monitoring and optimal treatment decisions.

## 2. Basic Principles and Applications of Liquid Biopsy 

Tumor cells may release multiple components into the circulation that can travel to distant organs throughout the body. These may include intact cancer cells and also tumor cell DNA and RNA, proteins, or exosomes that can be exploited as blood-circulating cancer biomarkers and may provide us with similar information to that of tissue biopsy, pinpointing the primary origin site and playing a key role in routine monitoring of tumor progression or treatment efficacy [5].

Given the increasing sensitivity of techniques that are used to study nucleic acids, it is nowadays possible to analyze tumor-released nucleic acids and blood-circulating cells. Genomic and epigenomic analysis of the circulating tumor DNA, for example, is an important novel tool regarding treatment. By using liquid biopsy (LB), clinicians can obtain data related to risk and disease prognosis and can predict chances of recurrence [9]. In more detail, LB is a minimally invasive procedure (e.g., venipuncture) that enables detection of several different analytes [6] in a variety of biological fluids to monitor disease progression [10,11]. Since most tumors encounter blood circulation, LB usually involves blood sampling. However, other body fluids, such as mucosa, fluid from pleural effusions, cerebrospinal fluid (CSF), and urine, may also be analyzed [12,13].

### 2.1. Analytes Detected in Biofluids by Liquid Biopsy

**Figure 1:**
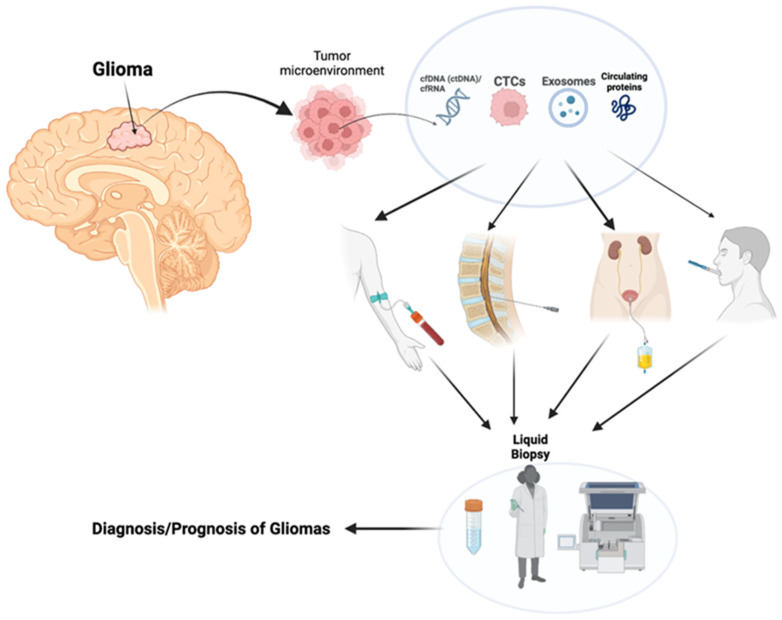
Detection of various tumor analytes in a variety of biofluids (CSF, urine, blood) with liquid biopsy. Several types of biofluids can be collected by using liquid biopsy and tumor-derived analytes (cfDNA, cfRNA, exosomes) measured using genomic processes to aid in tumor diagnosis, prognosis, and monitoring response to therapy (created with BioRender access on 24 June 2023).

In 1996, the National Comprehensive Cancer Network (NCCN) introduced several biomarkers into clinical practice, based on clinical and/or technical factors for diagnostic and prognostic purposes [14]. More recently, in 2011, the NCCN updated and established specific biomarkers as standard of care in some cancer types, including gliomas [15]. Analytes with a biomarker potential that can be detected through LB include cell-free DNA (cfDNA), cell-free RNA (cfRNA) and circulating proteins, circulating tumor cells (CTCs), and extracellular vesicles (EVs) such as exosomes, which can encapsule tumor DNA and other nanomolecules, such as mRNAs/miRNAs (Figure 1) [16,17].

#### 2.1.1. Cell-Free DNA (cfDNA), Cell-Free RNA (cfRNA), and Circulating Proteins

Circulating cfDNA refers to DNA fragments of the tumor cell DNA that can be detected in the circulation. Tumor cells that are being necrotized and/or undergoing apoptosis release cfDNA into the bloodstream, which is further digested by DNases or other enzymes [18]. Based on this evidence, patient’s blood may contain cfDNA that entails fragments of the circulating tumor DNA derived from tumor cells. This may be further analyzed on a molecular level to fully reveal the molecular profile of the cancer [19]. With regards to cfDNA characteristics, it has a half-life of almost two hours and it can be detected during that period after the isolation of the sample [20]. Based on the aforementioned data, cfDNA may be utilized as an indicator of tumor molecular dynamics in real time [18]. In patients with glioma, most of the cell-free DNA pool comprises non-tumor-derived cfDNA [21], most of which is believed to originate from various cellular events, including apoptosis, necrosis, and other cellular secretory processes. The specific mechanism of cfDNA release is dependent on a range of biological and environmental factors, including age, gender, body mass index, organ health, and the presence of infections or systemic inflammatory conditions [22,23].

Circulating tumor DNA (ctDNA) analysis involves the detection and analysis of DNA fragments released by tumor cells into the bloodstream. By isolating and sequencing ctDNA, specific genetic alterations associated with brain tumor can be identified and analyzed. cfDNA technologies can identify specific mutations in ctDNA that are indicative of brain neoplasm. These mutations may include single nucleotide variants (SNVs), insertions/deletions (indels), or larger structural variations, such as gene fusions or chromosomal rearrangements [24]. Copy number variation (CNV) analysis of cfDNA allows the detection of alterations in the DNA copy number, such as amplifications or deletions, which can provide insights into the genomic landscape of brain tumors [25,26]. These advanced technologies enable the real-time monitoring of tumor dynamics and treatment response by analyzing ctDNA at multiple time points during the course of treatment. This allows the detection of emerging genetic alterations and resistance mechanisms, guiding treatment decisions [27]. 

CSF has been proposed as the most suitable biological sample for cfDNA assessment in brain tumors; its collection, however, requires an invasive procedure that cannot be used routinely to monitor responses to therapy [28]. Therefore, there is a growing need for the utilization of alternative body fluids, such as blood, saliva, and urine, which can also be used in specific collection tubes and stabilization buffer/solutions, such as ethylenediaminetetraacetic acid (EDTA). 

Saliva, on the other hand, serves as a vital body fluid that possesses advantages over blood, primarily because it does not coagulate. This characteristic makes it easier to collect, transport, and store. However, it is important to note that saliva primarily comprises water, mucins, enzymes, and proteins, all of which can have an impact on downstream analyses. Additionally, salivary proteins, cfDNAs, and cfRNAs encapsulated within exosomes may rapidly degrade when they are exposed to an environment outside their natural milieu [29]. Furthermore, it is worth noting that saliva has a viscous nature and can contain food residues or other particles, which can pose challenges when analyzing cfDNAs, cfRNAs, or exosomes. However, it is important to consider that exosomes, being larger than proteins, may be inadvertently lost during the filtration process [30]. 

In addition, while urine holds great promise as a body fluid, our understanding of urinary cell-free Nucleic acids (cfNAs) and their processing methods remains limited. Urinary cfDNA, unlike cfDNA derived from blood, exhibits a shorter half-life [31] due to the elevated activity of DNase I and II [32,33]. It is crucial to inhibit DNases to prevent degradation and preserve the integrity of urinary cfDNA. One effective approach is to treat urine samples with EDTA and store them at −70 °C or lower temperatures for long-term preservation. Alternatively, sterile containers can be used to store samples at 4 °C or on ice, with immediate processing being preferable [34]. However, even under these conditions, there is a risk of cfDNA loss [35]. 

Similar to cfDNAs, cfRNAs are RNA fragments that are distinguished into coding and noncoding RNAs (ncRNAs) [36]. ncRNAs are further categorized by transcript length to smaller molecules that are less than 200 nucleotides in size and longer molecules that exceed 200 nucleotides. The group of small ncRNAs comprises microRNAs (miRNAs), short interfering RNAs (siRNAs), piwi-interacting RNAs (piRNAs), and small nucleolar RNAs (snoRNAs) [37,38]. 

The cf-ncRNAs molecules are characterized by high stability since they exist in vesicles or are associated with proteins [39]. They participate in cellular communication and may have a regulating role or alter tumor microenvironment, influencing tumor progress and invasion [40,41]. cf-ncRNAs present excellent biomarker candidates for diagnosing several diseases [41,42,43] but are also suitable for tumor screening and monitoring and studying resistance to therapy [44].

Finally, circulating proteins, oftentimes carried in exosomes and extracellular vesicles, are usually membrane receptors, receptor ligands, growth factors, or cytokines [45]. While numerous studies have successfully identified a range of protein cargo present in extracellular vesicles (EVs) derived from glioblastoma (GB), a comprehensive and detailed understanding of EV protein profiles remains yet to be explored [46].

#### 2.1.2. Circulating Tumor Cells (CTCs)

CTCs are cells that stem from a primary or secondary (metastatic) tumor. CTCs may be implicated in the metastatic process; however, cellular migration is extremely complex and its pathogenetic mechanisms need to be fully elucidated. Also, it remains to be clarified whether CTCs are derived from central tumor subpopulations or if they represent the entire tumor [47]. These cells enter the stream of different biological fluids such as blood, CSF, and urine. It is, however, important to mention that CTCs, in cases of brain tumors, are more conveniently collected from the CSF rather than from the bloodstream. Moreover, cisternal puncture seems to give more accurate results compared with lumbar puncture, although lumbar puncture seems to be less invasive and less dangerous regarding central nervous system complications. It also needs to be noted that CSF is recycled and fully exchanged every three to five days [48]. Therefore, a universally accepted method for CTC identification and collection needs yet to be discovered [49].

In this context, it is interesting to mention that circulating benign epithelial cells can also be found in inflammatory bowel diseases, which points out the need for a molecular characterization of CTC testing [50]. In a wide array of studies, CTCs seem to be useful as biomarkers for the prognosis of different cancer types, such as lung cancer, melanoma, etc. [51]. When it comes to brain tumors, and especially GB, it has been shown that CTCs can be detected in patients’ bloodstreams at a percentage of 20 to 40% [52,53].

#### 2.1.3. Extracellular Vesicles (EVs)/Exosomes and miRNAs

Exosomes were originally considered to be cellular waste products but are now known to serve as signaling vesicles between cells and coordinators of cellular communication through proteins and nucleotide transfer [54]. Exosome uptake depends on the acceptor cell type and primarily occurs through phago- or endocytosis [55,56]. 

Exosomes play a key role in disease pathophysiology, as well as cellular development, homeostasis, and immune response [57,58]. Cancer cells release a high number of exosomes into the extracellular space, the majority of which are made of functional biomolecules [59]. In this context, exosomes were initially researched as non-cellular therapeutic antigens for the development of vaccines against tumors or infectious diseases [60,61]. However, exosomes may also include miRNAs and other compounds that can mirror the progression of various brain diseases, which suggests that they can serve as representative tools for the molecular profile of the respective tumor [58,62] (Table 1).

## 3. Biomarkers Detected through Liquid Biopsy Analysis for the Diagnosis of Gliomas

The ability of a liquid biopsy to analyze tumor products that are found in body fluids has led to its increasing utilization in different types of tumors. This technique provides vital diagnostic and prognostic information, as well as real-time updates on tumor status. In the case of gliomas, the use of a liquid biopsy is extremely promising [74]. Below, we discuss additional cfDNA biomarkers detected in different studies of liquid biopsy analysis for the diagnosis of gliomas.

### 3.1. cfDNA, cfRNA, and Circulating Proteins in the Diagnosis of Gliomas

#### 3.1.1. cfDNA

LB has allowed the detection of cfDNA in cancer patients, as mentioned above, which has created the potential of its incorporation into clinical practice, with the goal of identifying both genetic and epigenetic alterations in tumors. cfDNA may be a suitable molecular marker, indicative of tumor status, which will enable disease monitoring and distinction between tumor-free individuals and brain tumor patients [75].

One study demonstrated that the serum of control subjects was consistently characterized by low levels of cfDNA, in contrast to GB patients who had higher cfDNA concentrations. This may suggest that cfDNA could serve as a useful biomarker for differentiating GB patients from healthy individuals and serve as an indicator for tumor progression [20]. 

cfDNA may also help in the quantitative measurement of genetic (e.g., *IDH* mutations) or epigenetic (*MGMT* methylation) alterations [75,76]. In the cfDNA derived from glioma patients, there are various genes and epigenetic alterations that can be detected and used as biomarkers to diagnose/monitor the disease. A prominent example is the *H3K27M* mutation characteristic of diffuse midline gliomas (DMG). In the study of Daphne Li et al., cfDNA was obtained from two types of specimens: H3.3K27M mutant and H3 wildtype (H3WT). The specimens included H3.3K27M tumor tissues (four samples), CSF (six samples), plasma (four samples), and human primary pediatric glioma cells. The researchers observed a sensitivity and specificity of 100% in detecting mutations in both matched DMG tissue and CSF samples [77]. Another study demonstrated that H3K27M was detected in the CSF and plasma of 88% of patients with DMG. Among the two, CSF exhibited the highest concentration of ctDNA [78]. 

Global DNA methylation (*LINE-1* or *L1*) may also play a pivotal role in the epigenetic status of glioma cells, enabling diagnosis through cfDNA analysis. The addition of a methyl group to cytosine, leading to the formation of 5-methylcytosine, is the most well-known mechanism of DNA modification through epigenetic processes, playing a significant role in the development and progression of cancer and, specifically, of gliomas [79,80]. The dysregulation of cells at a systemic level, often associated with tumor development, can result from either DNA hypomethylation or increased activity of *LINE-1* transposons [81]. The majority of transposition events occurring in the human genome are carried out by non-LTR (long-terminal repeat) retrotransposons, with long interspersed elements (LINEs) being the most prevalent. Of all LINEs, *LINE-1* accounts for approximately one-sixth of genomic transposition and exists as multiple copies in cell-free blood samples. Due to its abundance and unique characteristics, such as methylation status, it has the potential to serve as a valuable epigenetic biomarker for tumor detection in liquid biopsy samples [79].

Numerous studies have reported the detection of *LINE-1* retrotransposition events in various cell types, such as neural precursor cells (NPCs) derived from both human embryonic stem cells (ESCs) and fetal brain stem cells. Retrotransposition assays performed on these cell types confirmed the occurrence of *LINE-1* activity under both conditions [82]. 

In addition, quantitative polymerase chain reaction (qPCR) analysis has revealed a notably elevated number of *LINE-1* ORF2 copies in multiple regions of healthy adult human brain samples, indicating the presence of *LINE-1* activity within the brain. The utilization of retrotransposon capture sequencing (RC-Seq) also allowed the authors to identify somatic *LINE-1* insertions in protein-coding genes that were differentially expressed and actively functioning within the brain [83].

One study assessed *LINE-1* methylation levels through the quantitative bisulfite pyrosequencing of four CpG sites in healthy brain samples, as well as frozen tumor tissues, including grade 2/3 astrocytoma, primary/secondary GB, and grade 2/3 oligodendroglioma. A relatively higher degree of *LINE-1* methylation was observed in astrocytoma, oligodendroglioma, and oligoastrocytoma samples, while GB tumors exhibited the greatest variability in *LINE-1* methylation levels and the lowest *LINE-1* scores (indicating a global hypomethylation) compared with other subtypes. Additionally, the gliomas that were classified as having a class 1 pattern of gene methylation displayed significantly elevated *LINE-1* methylation levels [84]. 

Finally, normal brain tissues and LGGs exhibited higher *LINE-1* methylation levels compared with primary and secondary GBs and increased *LINE-1* methylation functions as a positive prognostic indicator in primary GBs. *LINE-1* methylation appeared to be present in all samples, including normal brain tissues, LGGs, and primary and secondary GBs [85]. This may suggest that *LINE-1* plays a key role in glioma diagnosis and/or prognosis.

Sabedot et al. utilized genome-wide DNA methylation profiling to detect unique methylation patterns in tumor tissue and cfDNA from glioma patients. They employed a score metric system named the “glioma-epigenetic liquid biopsy score”, or GeLB, to assess cfDNA methylome and diagnose gliomas. This non-invasive technique revealed a methylation signature in cfDNA associated with glioma presence and immune features. Independent cohort testing validated GeLB as a highly effective tool, with 100% sensitivity and 97.78% specificity for distinguishing glioma-positive and glioma-negative patients. GeLB score changes during surveillance reflected the clinicopathological conditions and treatment effects, suggesting its potential for diagnosis and monitoring of glioma patients [86].

Furthermore, Nassiri et al. showcased that plasma-based DNA methylation profiles exhibited distinctive signatures capable of effectively detecting and accurately distinguishing between common primary intracranial tumors. These tumors often shared cell-of-origin lineages, making them challenging to differentiate using standard imaging methods. The researchers utilized a technique called cell-free methylated DNA immunoprecipitation and high-throughput sequencing (cfMeDIP-seq) to successfully detect ctDNA derived from brain tumors [87].

Another study, by Martínez-Ricarte et al., performed genomic analysis of patients with diffuse gliomas that involved the examination of mutations in seven genes: *IDH1*, *IDH2*, *TP53*, *TERT*, *ATRX*, *H3F3A*, and *HIST1H3B* [88]. By analyzing ctDNA derived from CSF, the study successfully diagnosed diffuse gliomas and provided valuable support for surgical and clinical management decisions [88]. An additional study highlighted the importance of liquid biopsy in diagnosing and monitoring the evolving molecular landscape of tumors during treatment. The study aimed to identify *TERT* promoter mutations (C228T and C250T) in the cfDNA of glioma patients, providing evidence of the practical aspects of detecting circulating cfDNA *TERT* promoter mutations in patients with glioma, exhibiting clinically relevant sensitivity and specificity [89].

Fujioka et al. further developed a novel, highly sensitive, and specific molecular diagnostic method utilizing a chip-based digital PCR system to target ctDNA derived from CSF obtained from 11 patients with high-grade gliomas. The extracted ctDNA was analyzed for diagnostic point mutations in IDH1 R132H *TERT* promoter (C228T and C250T) and *H3F3A* (K27M). Diagnostic mutations were detected in tumor DNA samples from 28 out of 34 patients. Among these cases, precise molecular diagnoses were achieved using intracranial CSF in 20 patients, accounting for 71% of the cases [90].

The study of Husain et al. examined pre-radiotherapy cfDNA levels using next-generation sequencing (NGS) and compared the results to immunohistochemical (IHC) analysis, showing a high degree of accordance. This approach may prove beneficial in cases where surgery is not possible or in cases of adult diffuse glioma recurrence [91]. 

Finally, in the study of Palande et al., gene mutations and gene–gene fusions were detected in the circulating cfDNA of patients with GB. This study utilized the ChiTaRS 5.0 gene–gene fusion database to identify gene–gene fusions in cfDNA and tumor DNA samples. Notably, fusions involving *PDGFRA* were found in 44% of GB samples, which can be effectively targeted using tyrosine kinase inhibitors. Other gene fusion events, such as *BCR-ABL1*, *COL1A1-PDGFB*, *NIN-PDGFRB*, and *FGFR1-BCR*, were also detected in cfDNA and could be targeted with imatinib analogs. Additionally, *ROS1* fusions were identified in 8% of cfDNA samples and could potentially be targeted using specific drugs [92].

#### 3.1.2. cfRNA 

When it comes to cfRNAs and their use in gliomas, Dong et al. discovered that the serum of GB patients exhibited a significant decrease in 24 miRNAs and a substantial increase in 115 miRNAs, which was not observed in the serum of healthy controls [93]. Furthermore, Wang et al. identified three downregulated miRNAs, namely miR-128, miR-485-3p, and miR-342-3p, in patients compared with controls. These miRNAs demonstrated a correlation with GB grades and served as biomarkers for assessing tumor grading and monitoring treatment response [94].

MicroRNA-21 is the most extensively studied miRNA in cancer and its overexpression has been consistently observed both in tissue and plasma of GB patients. This elevated expression is closely linked to lower overall survival rates and higher tumor grading [95]. miR-21 functions as an anti-apoptotic factor in glioblastoma cell lines by exerting its effects through caspase inhibition. The suppression of miR-21 activity consequently impedes cell growth, enhances apoptosis, and diminishes the proliferation of GB cancer cells [95,96].

It is worth noting a study that identified TP73-AS1 as a clinically relevant long non-coding RNA (lncRNA) in GB. The researchers observed a significant upregulation of TP73-AS1 in primary GB samples. They provided evidence that TP73-AS1 serves as a robust prognostic biomarker, as this lncRNA promotes tumor aggressiveness and confers resistance to TMZ treatment in GB cancer stem cells [97].

In the study of Shen et al., increased levels of the long noncoding RNA HOX transcript antisense intergenic RNA (HOTAIR) and decreased levels of GAS5 were observed in the serum of GB patients. These expression patterns were associated with a reduced likelihood of 2-year survival [98]. Additionally, it was found that the transfer of long noncoding RNA HOTAIR via serum exosomes showed promising diagnostic value in GB [99].

#### 3.1.3. Circulating Proteins 

Given the remarkable patient and disease heterogeneity, as well as the broad protein involvement in multiple processes, it is difficult to find diagnostic plasma/serum markers for glioblastoma with sufficient clinical value. However, extensive research has identified the levels of several circulating proteins to be associated with the presence of GB. One example is the plasma glycoprotein, haptoglobin, which is an acute phase protein involved in the protection of tissue damage and oxidative stress [100]. Haptoglobin’s levels change during pathologies and, although its single estimation may not be specific and sensitive enough, several proteoforms of haptoglobin (α2 and β-chain) have been detected upregulated in GB patients’ plasma, indicating its potential use as a GB-specific blood biomarker [100]. 

In addition, a quantitative proteomic analysis showed that several plasma proteins, such as carnosinase 1 (CNDP1) that regulates carnosine levels, the inflammatory marker ferritin light chain (FTL), and the Ca^2+^ signaling protein S100A9, were found to be altered in GB tissues, with CNDP1 been suggested as a potential drug target. This confirms that plasma-based tests for initial diagnosis or recurrence monitoring can be highly useful for GB [101,102]. Moreover, the study of Pérez-Larraya et al. performed a comprehensive analysis of insulin-like growth factor binding protein 2 (IGFBP-2), the intermediate filament (IF) III protein GFAP, and the glycoprotein YKL-40 plasma levels as a supplementary diagnostic and prognostic tool [103]. This approach holds great promise, particularly for patients with inoperable brain lesions [103]. Interestingly, the presence of the glycoprotein’s fetuin-A auto-antibodies was also revealed as indicators of GB development [104]. Ectopic fetuin-A levels have been suggested to play a role in tumor progression, including GB. Therefore, evaluation of fetuin-A auto-antibodies in the serum of GB patients was proposed to potentially serve as a screening tool, presenting one of the earliest indicators for GB growth [104].

Naryzhny and colleagues aimed to identify common exosomal proteins in various cell lines and explore potential biomarkers for glioblastoma in exosomes. Through proteomic analysis of exosomes, they identified 133 proteins, such as pyruvate kinase PKM (KPYM), annexin A1 (ANXA1), transitional endoplasmic reticulum ATPase (TERA), alpha-enolase (ENOA), nucleophosmin (NPM), and cofilin (COF1), which were consistently present in all samples. The study established a correlation between certain proteins overexpressed in glial cells and their detection in exosomes, confirming the presence of numerous potential protein biomarkers for GB in exosomes [105]. Another investigation involving SWATH mass spectrometry and quantitative targeted absolute proteomics, where GB patients and healthy controls were studied. The findings revealed notable positive associations between leucine-rich alpha-2-glycoprotein (LRG1), complement component C9, and C-reactive protein (CRP) levels and tumor size [106]. In a meta-analysis conducted by Qin et al., a robust correlation was discovered between elevated YKL-40 expression and unfavorable overall survival among GB patients. This finding positions YKL-40 as a promising prognostic biomarker with significant disease-specific potential [107].

### 3.2. CTCs in Glioma Diagnosis

Numerous studies have confirmed the significant role of CTCs in CNS tumors. CTCs provide a less invasive way of obtaining tumor samples for cancer detection; certain CTC genotypes may indicate primary tumor progression and changes in malignant genetic information during disease relapse. GB has been reported to have a high prevalence of CTCs, which is estimated to be around 75% [108].

CTC concentrations have been utilized for the detection of various conditions. According to Santos et al., CTCs exhibit promising results in the early detection of colorectal cancer as they can be identified in the blood of patients who have recently developed the disease. As a result, CTC testing could be utilized to diagnose colorectal cancer [109].

When it comes to gliomas, the blood brain barrier (BBB) may restrict the quantity of CTCs which are found in the bloodstream. Despite this, circulating cells have been identified in glioma patients’ blood and several studies have reported the detection of CTCs in the bloodstream of glioma patients through the use of various isolation techniques. After using the technology of CTC-iChip to enrich CTCs and staining them with antibodies, such as SOX2, EGFR, tubulin b-3, c-MET, and A2B5, Sullivan et al. identified CTCs in GB patients’ blood with a frequency of 39%. They also discovered that the CTCs exhibited a mesenchymal molecular signature [48]. Muller et al. conducted a study where they employed Ficoll–Paque gradients through differential centrifugation, followed by GFAP staining, to identify 20.6% of CTCs [108]. In addition, Gao et al. demonstrated that CTCs were present in 77% of blood samples from patients with brain cancer and in 82% of patients with GB [52].

An interesting study by MacArthur et al. suggested that CTCs could also serve as a prognostic factor. Using density gradient centrifugation, they isolated CTCs and analyzed the activity of the telomerase enzyme in brain cancer patients. The study revealed that before radiotherapy treatment, CTCs were isolated in 72% of patients versus only in 8% after treatment [70]. Moreover, the anti-glioma aptamers Gli-233 and Gli-55 have been used to detect CTCs in LBs in order to increase diagnostic specificity [110]. Aptamers are nucleic acid molecules that bind exclusively to their targets, such as cancer-related proteins, with high affinity and selectivity [111]. They have therefore already been used in biosensing, bioregulation, and bioimaging as reliable recognition ligands [112]. Kichkailo et al. also demonstrated that aptamers may specifically bind to glial tumor cells for CNS tumor detection [110]. Although more research is necessary to uncover the full potential of CTCs in diagnosing gliomas and GB, their use as prognostic factors for gliomas is very promising. 

### 3.3. Exosomes in Glioma Diagnosis

Another important characteristic of GB cells is their ability to use invadopodia, through which they are capable of invading adjacent cells, specifically astrocytes. Invadopodia are GB cell membrane-derived extensions that adhere to neighboring tissues and promote the proteolytical degradation of the matrix, thus allowing invasion [113,114]. Several proteins excreted from GB-derived exosomes are linked to invadopodia biogenesis and subsequently GB-invasive potential, among which are annexin A1 (ANXA1), actin-related protein 3 (ACTR3), integrin β1 (ITGB1), calreticulin (CALR), and programmed cell death 6-interacting protein (PDCD6IP) [114]. Hallal et al. also observed the formation of podosomes in astrocytes and degradation of the matrix after their interaction with exosomes derived from GB cells, a process that appears to be favored by decreased p53 levels. Exosomes thereby exhibit carcinogenic potential and promote the neighboring astrocytes to become tumorigenic [115].

In this context, GB exosomes can be detected both in the blood and the CSF by using the minimally invasive technique of liquid biopsy. A robust number of tumor-derived exosomes may be found in the CSF. It is important to note that CSF is not contaminated with blood-related EVs, for example, platelet-derived exosomes. However, on the other hand, blood samples are more conveniently and less invasively collected compared with the CSF [113,116].

Furthermore, Shao et al. described how a nuclear magnetic resonance system can detect GB-expelled microvesicles in blood samples [117]. Studies have shown that exosome-carried molecules may promote tumor development and therapy resistance through the formation of a tumor-friendly microenvironment. Therefore, exosomes have been suggested as promising tools in GB diagnosis and prognosis, enabling a more specific characterization of the tumor [118,119].

Despite being carriers of many miRNA types, including miR-21/-29a/-221/-222, etc., as demonstrated in several studies (in vitro and microarray analyses), exosomes also play a key role in boosting proliferation and inhibiting GB cell apoptosis [58,120]. They could therefore be utilized as a promising delivery vehicle for tumor-suppressive miRNAs that target gliomas [121]. Approximately 1000 proteins have been identified in GB exosomes using mass spectrometry analysis; they mostly act as pro-angiogenic factors in normal endothelial cells of the brain, such as angiogenin, IL-6/-8, and tissue inhibitor of metalloproteinase-1 and 2 (TIMP-1, and TIMP-2), capable of promoting malignancy by causing hypoxia [122,123].

Exosomes may also transfer receptors with tumorigenic features, such as epidermal growth factor receptor vIII (EGFRvIII), human epidermal growth factor receptor 2 (HER2), and platelet-derived growth factor (PDGFR), which promote GB proliferation to healthy stromal cells [123]. Furthermore, exosomes deliver phosphatase and tensin homolog (PTEN), which is present in the nucleus or cytoplasm and whose absence has been associated with tumorigenesis [124] (Table 2). Ndfifip1 protein plays a major role in exosome internalization. Interestingly, the Ndfifip1 protein is repressed in GB; subsequently, the intranuclear concentration of PTEN is also suppressed, allowing tumor cells to proliferate and survive [124]. 

## 4. Combination of Radiogenomics and Liquid Biopsy for Glioma Diagnosis 

Radiogenomics is a field that integrates large amounts of quantitative data obtained from medical images with individual genomic phenotypes and uses deep learning techniques to develop a predictive model. This model is useful for stratifying patients, guiding therapeutic strategies, and assessing clinical outcomes [140].

**Figure 2:**
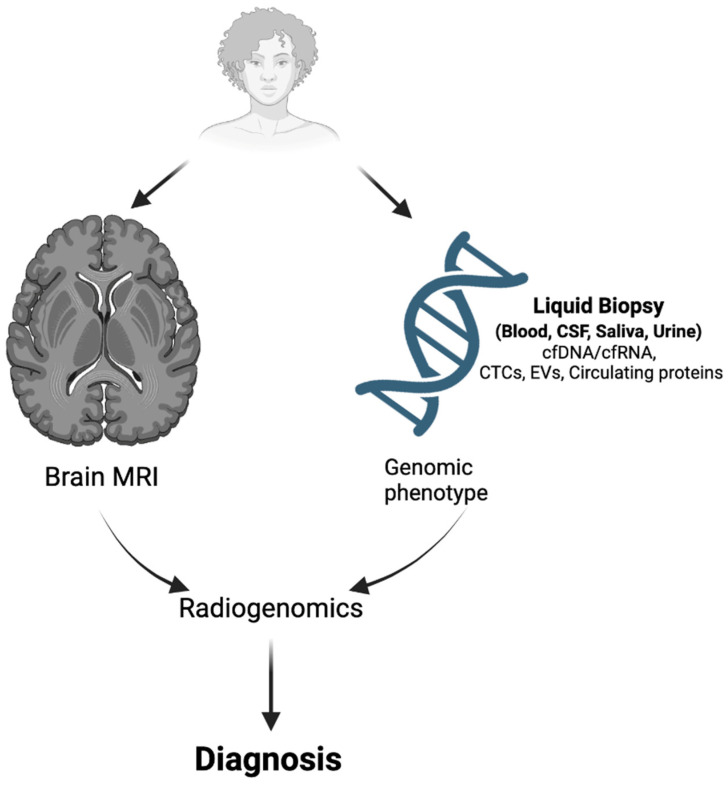
Impact of radiogenomics on diagnosis of brain tumors. Radiogenomics integrates large amounts of quantitative data obtained from medical images with individual genomic phenotypes from liquid biopsy analysis to enable efficient glioma diagnosis (created with BioRender, accessed 12 July 2023).

The combination of liquid biopsy and radiomics/radiogenomics can offer a promising avenue for non-invasive disease diagnosis and provide valuable insights for treatment planning (Figure 2). Both approaches have individually shown great potential, but their combination can lead to even more accurate and comprehensive diagnoses. The study of images using artificial intelligence (AI) and machine and deep learning techniques has progressed significantly in recent years. These methods involve an increasingly complex analysis of computational processes and imaging features, with the potential to accurately predict the molecular alterations necessary for correct disease diagnosis. As a result, they represent a promising approach for improving the accuracy and precision of glioma diagnosis [141,142]. In more detail, the field of radiogenomics offers a unique opportunity to predict genetic mutations, the status of molecular markers, and chromosomal aberrations through the analysis of imaging features. This approach utilizes imaging data as a substitute for the presence of genetic alterations, providing a non-invasive and efficient way of diagnosing and monitoring gliomas [143].

Radiogenomics has already been employed in other forms of cancer, such as breast [144] and lung cancer [145]. These studies have demonstrated the potential of radiogenomics to accurately predict the genetic characteristics of tumors through the analysis of imaging data. The extension of this approach to gliomas represents a significant opportunity to improve the accuracy and efficiency of non-invasive diagnosis and monitoring.

In the study of Li Y et al., for example, that used MRI radiomics (machine learning), the authors were able to predict ATRX mutations in low-grade gliomas [146]. In another study, the same mutations were identified in gliomas with deep learning, with 94% sensitivity and 92% specificity [147]. Diffuse midline gliomas with H3F3A histone mutations have greater enhancement in the T2 sequence, while tumors without the mutation have a poor identification of the non-contrast-enhancing tumor (NCET) margin. Furthermore, non-mutated tumors present with sound edema and cortical invasion [148]. A study of radiomics analysis (multiparameter MRI) managed to identify that tumors harboring TERT mutations have more necrotic areas, since TERT mutations suggest a high-grade profile [149].

Moon et al. also identified several characteristics of high-grade gliomas relative to *MGMT* promoter methylation [150]. When it comes to other brain tumors and, particularly, oligodendrogliomas, which are subcategorized to IDH-mutant and 1p/19q co-deleted [151], studies have been able to predict the 1p/19q codeletion with high specificity and sensitivity using, for example, a textural analysis of the T2 sequence [152,153,154]. It therefore becomes evident that the combination of liquid biopsy with the genomics component of radiogenomics profiling represents a promising approach to revolutionize cancer treatment. This combination may have the potential to facilitate early diagnosis, provide more accurate prognostic assessment, and enable real-time disease monitoring, all while minimizing invasiveness. Furthermore, this personalized approach to non-invasive brain tumor diagnosis could pave the way for precision medicine, tailored to the individual needs of each patient.

## 5. Conclusions: Future Perspectives

Significant advances have been made in the field of liquid biopsy in the last 20 years, moving from basic research to clinical applications in certain tumors such as lung, prostate and breast cancer, and melanoma. However, its integration into routine medical practices outside of academic hospitals remains limited. Despite various studies demonstrating promising results in various tumor types and clinical applications, including disease diagnosis, prognosis, monitoring, and therapeutic responses, the existence of standardized methods and useful genetic markers is still lacking. The process of biofluid collection, choice of biomarker, and detection strategy need to be extensively studied in future clinical trials in order to allow for the incorporation of LB into clinical practice.

Moreover, a series of factors may influence the utility, sensitivity, and specificity of different LB biomarkers, such as tumor subtype (GB vs. lower-grade gliomas for example), tumor size and location, extent of BBB disruption, and tumor stage. In more detail, the value of each biomarker will vary based on the different stages of the disease, such as during the initial diagnosis, patient surveillance with stable disease, active disease progression, or disease recurrence, so that biomarker combinations may need to be considered [155]. Moreover, the ideal site for biomarker collection needs to be specified for each patient and each tumor type, as well as the best method in cases of CSF collection, namely lumbar versus cisternal, as mentioned above. It is also necessary to find circulating biomarkers that are specific for each tumor type, as many are not entirely specific to GB and do not allow for the discrimination between different tumor types in patients with similar symptoms or subcategories of the same tumor [156]. 

Altogether, the utilization of liquid biopsy and radiogenomics presents a significant opportunity to achieve non-invasive brain tumor diagnoses and successfully identify their molecular alterations. If the abovementioned issues are overcome, analysis of tumor constituents in bodily fluids and utilization of imaging data to predict genetic abnormalities by using these techniques can provide critical insights into the nature and behavior of CNS tumors, thereby allowing for more effective treatment planning and monitoring.

## Figures and Tables

**Table 1:** Main analytes detected by liquid biopsies.

LB Sample	Sample Source	Biomarker Isolation Technique	Benefit	Reference
cfDNA (ctDNA)	Blood, CSF, Saliva	MS/digital PCR, sequencing, LOH ddPCR, MAF, TAS/WES, NGS	Molecular diagnosis, tumor growth, and therapy response monitoring	[27,28,30,63,64,65,66,67]
cfRNA	Blood, CSF	Microarray, qPCR	Tumor growth, therapy response monitoring, and molecular diagnosis	[41,42,43,44,62]
Circulating proteins	Blood, Urine, CSF, Saliva	qPCR, NGS, microarray	Diagnosis and prognosis	[16,45,46]
CTCs	Blood, CSF	EpCAM Immunomagnetic isolation, FISH, density gradient centrifugation/ telomerase activity	Molecular diagnosis, tumor Growth, and therapy response monitoring	[52,53,68,69,70]
EVs	Blood, Saliva	qPCR, NGS, microarray	Molecular diagnosis and disease prognosis	[30,58,71,72,73]

MS PCR—methylation-specific PCR; NGS—next generation sequencing; TAS—targeted analysis sequencing; WES—whole exome sequencing; WGS—whole genome sequencing, ddPCR—droplet digital PCR; MAF—mutant allelic frequency; EpCAM—epithelial cell adhesion molecule; FISH—fluorescence in situ hybridization; qPCR—quantitative polymerase chain reaction.

**Table 2:** Glioma-derived exosomes biomarkers.

Sample	Cargo (Biomarker)	Model	Benefit	Reference
CSF/Plasma	miRNA-21	Patients with glioma (grade I to IV)	Characterization of stable versus progressive disease	[120,125,126]
CSF	miR-21/miR-103/miR-24/miR-125	Patients with GB	Disease diagnosis	[126,127]
CSF	miR-151a	Patients with GB who received TMZ treatment	Prediction of treatment response	[128]
Blood	EGFR/EGFRvIII	In vitro (μNMR)	Characterization of molecular status	[123,129]
Blood	PTRF	In vivo mice xenograft	Disease diagnosis	[130]
Blood	MGMT, APNG	Patients with GB (microfluidic chip)	Disease prognosis	[76,131]
Serum	miR-320/miR-574 3p/RNU6-1	Patients with GB	Disease diagnosis	[132]
Serum	miR-301a	In vitro	Disease prognosis	[133]
Serum	miR-15b-5p, miR-16-5p, miR-19a-3p, miR-19b-3p, miR-20a-5p, miR-106a-5p, miR-130-3p, miR-181b-5p, miR-208a-3p	Astrocytoma (grade II to IV) (TaqMan low-density array)	Disease prognosis	[134,135]
Serum	miR-497, miR-125b	Patients with GB	Disease prognosis	[136]
Plasma	miR-221/222	Patients with high-grade gliomas	Disease diagnosis, Prediction of poorer prognosis and response to treatment	[137]
Plasma	PDGFR, CAV1, IL-8	In vivo glioma mice xenograft	Disease diagnosis and prognosis	[138]
Blood	miR-100	Patients with GB	Disease diagnosis	[139]

## Data Availability

Not applicable.
